# Problems of Venereal Disease

**Published:** 1920-10-09

**Authors:** 


### THE PUBLIC WELL-BEING.

### Problems of Venereal Disease.

### A VOLUNTARY COMMITTEE'S REPORT DISCUSSED.

WE have read with interest the very frank and exhaustive report just issued in book form by an influential Committee appointed, at the request of the Association for Moral and Social Hygiene, to inquire into the conditions of sexual morality in this country, with special reference to the improvement of the laws and their administration.

The inquiry was drawn up on independent lines, and the report generally takes the standpoint that the moral reformation of individuals is more likely to be brought about by voluntary methods than by compulsion, and therefore the Government is urged to improve and co-ordinate existing agencies, whether official or voluntary. It is clear to them, say the Committee, that in dealing with relations so intimate, so sensitive, and so individual as those of sex "the attempt to suppress vice by penal legislation directed against it can accomplish very little good. Such legislation has, on the contrary, increased immorality by taking away the hope and the opportunity of recovery. Indirectly, by providing means of recovery and by the improvement of the general conditions of life, the Government can do much. Individual and associated understanding and effort can do no more." This sentence supplies the keynote of the report.

### No Evidence of Increase.

The most important section is that which deals with venereal disease. "Despite the inadequate provision during the last half-century for treating venereal disease, there is no evidence in the Report of the Royal Commission that there has been any increase in these diseases in that period. On the contrary, such statistics as are available indicate a substantial decrease. The Committee think that the Royal Commission's estimate that the number of persons who have been affected with syphilis, acquired or congenital, cannot fall below ten per cent. of the whole population in the large cities, and that the percentage infected with gonorrhœa must greatly exceed this proportion is "pure guess-work, and probably much exaggerated." As to this it can only be emphasised that the Committee's information on this point cannot be considered for a moment as sufficient to compare with that of the Royal Commission, nor does its composition entitle its conclusion to equal respect.

The Committee add that they do not wish to minimise the seriousness of the situation, and they realise that "there has been some increase of disease consequent on the war"; but they do not think the situation so alarming as to justify "the hasty adoption of new and experimental measures, especially if such measures involve any of those objectionable features which were at the foundations of the vice-regulation systems, which have been tried in this country and elsewhere and have invariably failed."

Steps have already been taken by many local authorities to carry out the recommendations of the Ministry of Health, which contain no element of compulsion, but so short a time has elapsed since the adoption of the system "that it is obviously impossible to say that it has at present had a fair trial." "We accordingly think it necessary to view with grave suspicion the various proposals which have since been made for dealing with this matter by legislative or administrative action, since any coercive or ill-considered methods may endanger the prospects of the voluntary system before it has had time to come into fully successful operation."

### The Committee and "Quacks."

It is rather surprising to find that the Committee strongly object to Provision 52 of the Venereal Diseases Act of 1917 as to treatment and advice "because it goes beyond the penalisation of quacks, since it also prevents any pharmaceutical chemist from recommending or offering for sale any drug, etc., for the treatment of venereal disease." "A chemist is not prohibited from prescribing for any other disease" (it may be pointed out, however, that this comment may not hold good much longer), and the Committee doubt the expediency of treating venereal diseases, in this respect, differently from other diseases. They recommend, therefore, the repeal of the provision, being of opinion that the prohibition of treating any disease except by qualified practitioner "is inconsistent with the freedom of research which is essential to scientific progress"—a phrase which we cannot help thinking is open to much misconception and criticism.

### Prophylaxis Condemned.

The Committee summarise a good deal of evidence against the prophylactic system, and quote approvingly a memorandum from the Medical Women's Federation which impresses the belief that if the "packet" system were generally adopted "safety from infection would not be attained, while moral degeneration and the excesses would rot the very foundation of society." The Committee entirely concur in this view that no approval or encouragement should be given by any public authority to the policy advocated by the Society for the Prevention of Venereal Disease, and "profoundly regret" that measures have already been taken by the Manchester Corporation for recommending the Society's leaflet by means of posters in the public lavatories.

Notification also receives scant favour, the investigators being convinced that it would be quite impracticable to enforce any legislation, based on the Australian model, impartially and universally; and they are of opinion that any partial application of such measures to particular classes of the population, *e.g.*, to prostitutes or to prisoners, or to men or women who, owing to poverty, are obliged to enter hospitals or other institutions, would be not

**THE PUBLIC WELL-BEING**—(*continued*).

only unjust, but ineffective as a means of reducing the prevalence of the disease.

With regard to searching the transmission of venereal disease the Committee do not give very clear guidance. It is important, they say, to distinguish between four classes of cases—namely, accidental infection, infection of children, infection in marriage, and infection in promiscuous intercourse, which vary so widely in character and the circumstances that "it seems improper to subject them to one and the same penalty." Even if accidental infection should be dealt with ("if at all") by public health legislation, cases of infection in marriage should be met by allowing release from marriage, either by separation order or divorce, or by a decree of nullity. As to infection in promiscuous intercourse—the most frequent source of infection—such intercourse is in itself unhygienic, and the aim and object of hygienic reform should be to discourage all such intercourse. "People indulging in it ought to understand clearly that they are deliberately exposing themselves to the possibility of venereal infection, and do so at their own risk." Any legislative or administrative measures which tend to obscure this fact by offering a false security "are likely, instead of diminishing the prevalence of the disease, to increase it by increasing the amount of indulgence."

What, then, do the Committee propose, since they condemn or discourage so many practical proposals? They propose instruction in moral conduct among all classes, the teaching of a single standard of morals on both sides, and the development of a higher moral standard throughout the community. On the whole, one reads the report with some disappointment on account of the indefiniteness of its conclusions.

